# Brain Plasticity in Patients with Spinal Cord Injuries: A Systematic Review

**DOI:** 10.3390/ijms25042224

**Published:** 2024-02-13

**Authors:** Andrea Calderone, Davide Cardile, Rosaria De Luca, Angelo Quartarone, Francesco Corallo, Rocco Salvatore Calabrò

**Affiliations:** 1 Graduate School of Health Psychology, Department of Clinical and Experimental Medicine, University of Messina, 98122 Messina, Italy; andrea.calderone95@gmail.com; 2 IRCCS Centro Neurolesi Bonino-Pulejo, S.S. 113 Via Palermo, C.da Casazza, 98124 Messina, Italy

**Keywords:** spinal cord injury, brain plasticity, neurorehabilitation

## Abstract

A spinal cord injury (SCI) causes changes in brain structure and brain function due to the direct effects of nerve damage, secondary mechanisms, and long-term effects of the injury, such as paralysis and neuropathic pain (NP). Recovery takes place over weeks to months, which is a time frame well beyond the duration of spinal shock and is the phase in which the spinal cord remains unstimulated below the level of injury and is associated with adaptations occurring throughout the nervous system, often referred to as neuronal plasticity. Such changes occur at different anatomical sites and also at different physiological and molecular biological levels. This review aims to investigate brain plasticity in patients with SCIs and its influence on the rehabilitation process. Studies were identified from an online search of the PubMed, Web of Science, and Scopus databases. Studies published between 2013 and 2023 were selected. This review has been registered on OSF under (n) 9QP45. We found that neuroplasticity can affect the sensory-motor network, and different protocols or rehabilitation interventions can activate this process in different ways. Exercise rehabilitation training in humans with SCIs can elicit white matter plasticity in the form of increased myelin water content. This review has demonstrated that SCI patients may experience plastic changes either spontaneously or as a result of specific neurorehabilitation training, which may lead to positive outcomes in functional recovery. Clinical and experimental evidence convincingly displays that plasticity occurs in the adult CNS through a variety of events following traumatic or non-traumatic SCI. Furthermore, efficacy-based, pharmacological, and genetic approaches, alone or in combination, are increasingly effective in promoting plasticity.

## 1. Introduction

The changes in brain structure and function caused by SCIs are a result of the direct effects, secondary mechanisms, and long-term consequences. These effects include paralysis and NP [1]. According to the World Health Organization, every year on average, between 250,000 and 500,000 people suffer from SCIs [2]. The consequence of this medical ailment is immediate and severe on motor control and many important physiological functions. In the last century, there has been a shift towards a better understanding of the mechanisms of injury in the clinical treatment of SCI. Functional improvement and reduced morbidity have been observed in patients with SCIs, resulting from surgical intervention, supportive care, and rehabilitation. Many disabilities are permanent due to the limited capacity of the central nervous system (CNS) to repair itself after injury. Half of those affected remain paralyzed, suffer permanent disability, and have a life expectancy of several decades [3,4]. The recovery of motor, sensory, and autonomic functions after SCIs in both humans and animals is either a spontaneous or delayed response to the severity of the injury. At the same time, potentially harmful and painful experiences may take place [5]. A summary of the mechanisms behind these different functional changes is given by the term plasticity. Although it may take weeks or months, the recovery time is considerably longer than the spinal shock phase [6]. The nervous system’s adaptations are often associated with neuroplasticity, a stage in which the spinal cord remains unaltered below the injury point [7,8,9,10,11]. These changes take place at varying locations in anatomical contexts and at various physiological and molecular biological levels. The sensorimotor cortex, brainstem, and spinal cord in humans and animals have been found to exhibit plasticity after SCIs [12,13,14,15,16,17,18,19,20,21]. Also, above and below the level of injury in the spinal cord, network reorganization has been detected [9,16,22,23,24,25,26,27,28]. Plasticity can be influenced by a variety of mechanisms, including the growth of new axial branches [16,28,29,30], synaptic remodeling [31,32], and changes in neuronal characteristics such as sustained inward currents [33]. For example, most studies on axon regeneration have investigated the plasticity of motor fibers, particularly the unilateral corticospinal tract (CST), which regulates fine motor movements [34] usually by monitoring anterograde motor pathways using adeno-associated viruses or injections of biotinylated dextranamine. After an incomplete SCI, CST fibers can either regenerate or sprout or at least reorganize and recover functional motor circuits alongside neurons in the spinal cord [35]. The control of movement is not limited to these CST fibers but also extends through various other pathways such as the rubrospinal, tectospinal, and even the reticulospinal tracts (RSTs) [36]. One of the most intriguing pathways to control automatic rhythmic movements, including locomotion, is RSTs, which is also of significant interest due to its use in the projection of a central pattern generator. RSTs neurons are mainly glutamatergic and play different or opposite roles during movement and are composed of several cells. They are composed of subpopulations [37], and few studies have examined RSTs’ plasticity. However, RSTs have been shown to sprout below the lesion site after an incomplete SCI [38]. Other studies have instead shown that RSTs can spontaneously sprout in the spinal or caudal to the spinal lesion [38]. Injured and uninjured corticospinal axons showed sprouting in the days and weeks after injury, both locally and globally. Corticospinal sprouting and synaptic connections with cervical propriospinalneurons, which projected to adjacent or distal spinal segments, were established after the thoracic spinal cord’s dorsal CST lesion [39]. Also, the fast cortical organization that follows minor lesions is believed to be based on a closely linked network of various subregions of the primary motor cortex (M1). The horizontal pathways are not evenly spaced within the M1, with some subregions being densely populated and others lacking [40,41]. The connections among the cortical, subcortical, and spinal motor systems suggest that plasticity occurring at one level may influence changes occurring at other levels. This has been demonstrated by changes in motor maps in the M1 following a SCI. Animal models of SCIs have been found to exhibit spontaneous circuit reorganization, which has led to the development of useful repair strategies. During the process of incomplete injury, intraspinal projection neurons generate new “detour circuits” that transmit information onto the spinal cord from above to below the injury [42,43]. The corticospinal and reticulospinal neurons, which are connected to areas beneath the lesion, are proliferated by intrasolar relay neurons [18,24]. Following SCIs in animal models, there was a marked degree of restructuring in all the residual descending system spinal cords (axons) [44,45,46]. Non-human primates have a distinct set of corticospinal axillary cells that function as collateral axels, and they descend into the contralateral white matter after undergoing lateral hemisectioning [47,48]. These findings are similar to those of the reticulospinal, rubrospinal, and serotonergic descending systems [18,49,50]. According to some animal models motor recovery is linked to the corticospinal and reticulospinal tracts [51,52,53]. Motor cortical output is reassigned to spinal circuits via the plasticity found in the spinal cord. The motor cortex gains a crucial role in movement following injury through this remarkable reorganization. The mediating of recovery is also facilitated by intraspinal projection circuits [44,54,55]. The spinal cord’s intricate web of ascension and decomposition can serve as an ideal target for SCI repair [56]. Also, in non-human primates, intraspinal relay circuits can facilitate spontaneous fine finger movements after the loss of direct corticospinal tract input to cervical motor centers [57]. Taken together, these observations suggest either promoting the spontaneous formation of relay circuits after an incomplete SCI or allowing axonal regrowth across anatomically complete SCI lesions, even over short distances. SCIs disrupts spinal circuits, but they still receive and interpret sensory information. Intact sensory afferents below lesions control motor behaviors and steer beneficial and detrimental changes. Proprioceptive organ inputs guide the reorganization of supraspinal and intraspinal projection neurons, restoring locomotion after SCI. The absence of functional proprioceptive circuits in mice with SCIs results in defects in the relocation of the descending projection circuit, which renders recovery impossible. Sensory afferents are abnormally sprouted to available synaptic targets when the depletion of synapses from descending pathways occurs [58,59]. The absence of signals controlling homeostatic plasticity often leads to a decrease in neuronal function below a SCI, as evidenced by animal models of SCIs [58]. Moreover, midbrain neurons in rats that are exposed to reduced oxygen levels (intermittent hypoxia) experience an increase in serotonergic raphe neurotransmitters activated by this transient exposure. This triggers the activation of brain-derived neurotrophin-mediated pathways. Together, they produce serotonin and induce the dependent plasticity of spinal processes [60]. The improvement of gait in people with incomplete paraplegia has been demonstrated by clinical trials that combine rehabilitation with repeated intermittent hypoxia [61]. In various animal models, cell grafts that repopulate non-neural cores with growth-supportive neuroglia, such as Schwann cells [62,63] or astroglia [64,65], facilitate the growth of host axons across non-neural lesion cores and improve the functionality of residual fibers [66]. A clinical trial was conducted to evaluate the safety and effectiveness of autologous Schwann cell transplantation for SCIs [67]. To facilitate the development of relay circuits, a different intraspinal source of neurons can be transplanted as an alternative method. Neural precursor cell grafts that are formed caudally, in the presence of growth factors and supporting substrates, not only survive in a harsh non-neoplastic environment but also regenerate the center of their lesion. They develop into a cluster of neurons and glial cells that transmit transient axial projections and host tissues in rostral and rodent lesions [68]. Myelin can promote axon growth in transplanted cells by activating their cell adhesion molecule nerve growth regulator 1 (NEGR1), which is one of the factors that facilitates these interactions. Also, transplanted neurons release various growth factors that draw in a significant number of synaptic impulses from all surrounding systems to stimulate the robust regeneration of refractory corticospinal tracts [69]. Neutral relay circuits are formed through the formation of neural precursor grafts, which restore partial communication across anatomically intact SCIs [70]. After traumatic or non-traumatic SCIs, the careful investigation of the level and extent of lesions and secondary course evolution is essential for treatment planning. Magnetic resonance imaging (MRI), together with clinical features, has become the gold standard to guide optimal treatment after SCIs [71]. MRIs clearly show the often-devastating morphologic sequelae of typical spinal cord lesions. This instrument may be very useful to see the changes in brain plasticity in action over time, thanks to a large variety of MRI techniques [72]. Quantitative MRI studies have revealed that traumatic SCI leads to a series of secondary neurodegenerative disorders, including demyelination and iron deposition in the brain [73], as compared to standard scans [74]. It is currently believed that the development of NP is linked to fluctuations in macrostructural changes observed in both the brain and medulla. The preservation of macrostructural tissue (ventral tissue bridge) and the growth and maintenance of NP after SCIs may be connected [75]. Plasticity activity is a key factor in altering the structure of myelin, according to recent research. Kyathanahally et al, through multiparametric mapping and quantitative MRIs of SCIs, found that the myelin-sensitive and iron-sensitivity differences associated with NPs along the nociceptive pathway were lower and higher [76]. In humans, there has been evidence of NP-related modifications in the periaqueductal gray (PAG) ultrastructure, which suggests that these modifications are important for NP after SCIs. Combined with microstructural changes in the injured spinal cord, these results are consistent with the concept of dysfunction of the bulbospinal loop, which is a critical circuit in the descending pain modulation system [77]. Along with trauma-related changes, the volumes of the cervical spinal cord, thalamus, and primary somatosensory cortex increase and decrease after SCIs. The pathophysiology of this process involves chronic ectopic electrical activity in the spinothalamic tract’s remaining axons [78], an increase in sodium channel upregulation in nociceptive neurons [79], and increased proinflammatory cytokines [80]. Further evidence of the impairment of descending pain modulation systems suggests that other important components of this network, namely circuits in the anterior cingulate cortex (increased iron accumulation) and dorsolateral prefrontal cortex (decreased myelin content), can be obtained from changes in the microstructure [81]. MRI studies using animal models, from a rehabilitation perspective, have shown that primates (monkeys and humans) can achieve significantly better locomotor recovery than rodents after unilateral SCIs through a different circuit. Structural reorganization, such as synaptic remodeling, axonal dendritic growth, and circuit reorganization in the supralesional and sublesional spinal cord, can restore function after an injury [82,83,84].To understand the effects of brain plasticity after SCIs from a functional point of view, scales such as the Functional Index Measure [85] and more recent and diagnosis-specific measures such as the Spinal Cord Independence Measure, which fit well with the overall assessment of patient functionality, can be used [86,87]. Additionally, various analyses on different potentials have been developed to investigate how different types of neural signals are transmitted through the same pathway (including motor control) in SCIs. By using test methods, it is possible to measure the accuracy and completeness of signal transmission and signal processing at all SCI levels. Most of these tests are based on electrophysiological data that directly measure the neurophysiology of the human spine. Motor evoked potentials (MEP), transcranial magnetic stimulation (TMS), somatosensory evoked potentials, contact thermal evoked potentials, quantitative sensory tests, and laser evoked potential all characterize the ability of signals to travel up and down the spinal cord [88,89,90,91,92,93,94,95]. A summary and brief description of the evoked potentials and tests are shown in Table 1.

The recovery of motor skills requires brain plasticity, which is a crucial aspect of neurorehabilitation for SCIs, regardless of the severity and duration of harm [96]. Neuroplasticity and neural repair are now being used to restore functional motor skills, rather than solely focusing on compensating for SCIs. This is the focus of modern neurorehabilitation. In this context, after an incomplete SCI, the CNS can regain motor function via functional training on a treadmill combined with partial body weight support [97,98]. After over a century of studying how motor control is accomplished, it has been established that the lumbosacral spinal cord contains the essential neural circuits responsible for efficient stepping patterns in mammals. [99,100]. These spinal motor circuits appear to play an important role in the ability to step in animal models and humans with SCIs. When the spinal neural circuits are activated below the lesion level, it is believed that proper afferent input is necessary for maintaining functional recovery after SCIs [101]. In contrast, typical motor impairments seen after SCIs, such as spastic motor impairment, result from the unavailability of afferent input combined with secondary compensatory mechanisms [102]. The neural networks underlying the generation of motor patterns in cats [103] and humans [59,104] are surprisingly flexible after SCIs. Therefore, rehabilitation interventions after SCIs should not focus on improving isolated clinical signs, such as muscle tone or reflex excitability, but on exploiting the plasticity of the neural circuitry at the supraspinal and/or spinal levels.

This scoping review aims to investigate brain plasticity in patients with SCIs and its influence on the rehabilitation process.

## 2. Methods

### 2.1. Search Strategy

**Figure 1:**
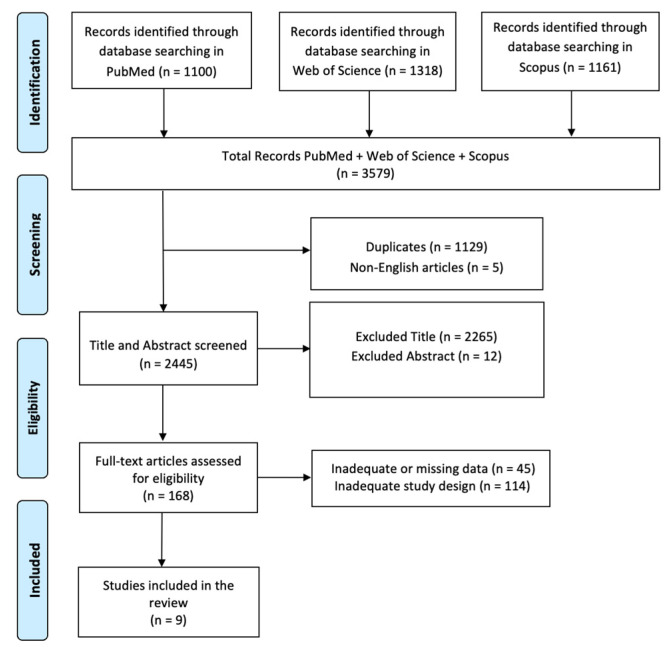
PRISMA 2020 flow diagram of evaluated studies.

A literature search was conducted via PubMed, Web of Science, and Scopus, and it was carried out for articles using the following search keyword terms: (All Fields: “Spinal Cord Injury”) AND (All Fields: “Brain Plasticity”); 2013–2023 was the search time range. We adopted the PRISMA (Preferred Reporting Items for Systematic Reviews and Meta-Analyses) flow diagram to describe the sequence of steps (identification, screening, eligibility, and inclusion) for the collection and determination of qualified studies as shown in Figure 1. Titles and abstracts were independently scanned and retrieved from database searches. The suitability of the article was then assessed according to the defined inclusion criteria. Ultimately, we selected all titles and abstracts that met the criteria for inclusion in the full text. To prevent bias, several expert teams worked together to choose articles, analyze data independently, and discuss any differences with each other. Disagreements between reviewers were resolved by consensus. This review has been registered on OSF under (n) 9QP45.

### 2.2. PICO Evaluation

We defined our combination of search terms using a PICO (population, intervention, comparison, outcome) model. The population was limited to patients with moderate to severe SCIs. The intervention included all studies, rehabilitation approaches, and assessment tools to measure and understand brain plasticity and its influence on the rehabilitation process. The comparison was evaluated considering the different instruments and interventions that produced some neural plasticity effects in patients with SCIs both before and during a psychological and motor rehabilitation process. And the results included any improvements in the neural activity, plasticity, and functioning of these patients during the rehabilitation process.

### 2.3. Inclusion Criteria

A study was included if it described or investigated the brain plasticity of a patient with a SCI and its influence on the rehabilitation process. The review included only articles written in English. Studies describing or investigating the functional assessment of these patients were also included. We only included studies conducted in human populations and published in English that met the following criteria: (i) original or protocol studies of any type and (ii) articles that presented the brain plasticity topic in patients with SCIs and its influence on the rehabilitation process.

### 2.4. Exclusion Criteria

A study was excluded if there was a lack of data or information on the description of the brain plasticity of patients with SCIs and its influence on the rehabilitation process. Reference lists were added as needed, but systematic reviews and integrated or narrative ones were not included. All articles written in languages other than English were excluded.

## 3. Results and Discussion

In total, 3579 articles were found: 1129 articles were removed due to duplication after screening; 5 articles were excluded because they were not published in English; 2277 articles were excluded based on title and abstract screening; finally, 159 articles were removed based on screening for inadequate study designs and untraceable articles (Figure 1). Nine research articles met the inclusion criteria and were therefore included in the review. A survey of these studies is shown in Table 2.

The articles described in this review investigated the brain plasticity of patients with SCIs and its influence on the rehabilitation process. The cortical sensory-motor plasticity in patients with SCIs was analyzed in three articles [105,106,107]. The rehabilitation effects on neural networks and functional recovery were described in six articles [108,109,110,111,112,113].

### 3.1. Cortical Sensory-Motor Plasticity in Patient with SCI

The sensory-motor network includes functional areas of the M1, cingulate cortex, premotor cortex, and supplementary motor cortex. Neuroplasticity can affect these areas after SCIs and during rehabilitation. In a study, paired-associate stimulation (PAS) and TMS were used in combination to achieve remarkably high corticospinal excitability in healthy volunteers and SCI patients, with motor recovery lasting up to 30 min. Despite having slightly higher resting motor thresholds in both groups of SCI patients, no significant differences were observed. The MEP amplitude of SCI patients with poor functional recovery showed no significant increase [105]. In a randomized trial of 20 participants, it was demonstrated that Hebbian stimulation can produce greater improvements in walking speed and corticospinal function than sham stimulation. As a result, prospective study participants showed improvements in grip and gait, corticospinal function, and quality of life measures, with further improvements noted with each successive session that continued for nine months after treatment. After 40 sessions, there was a positive correlation between MEP changes in each muscle and baseline MEP size. The results showed that patients with larger MEPs at baseline had broader residual cortical movements, and MEP size increased to varying degrees [106]. Another randomized controlled trial showed that the form of gait training had different effects on the cutaneomuscular reflex. Walking with endurance training (not precision training) resulted in increased inhibition of the soleus muscle’s reflexes. None of the training systematically changed clonus. Even so, clonus was strongly associated with the ability to walk, and improved gait corresponded to a lower clonus. A weaker association between improved gait and increased cutaneous muscle reflex inhibition was observed in individuals who underwent endurance training. The results indicate that certain types of gait training decrease reflex excitability, and some aspects of reduced reflex compatibility are associated with better gaits [107].

### 3.2. Rehabilitation, Neural Network, and Functional Recovery in SCI

Providing rehabilitation and protocol interventions for such patients can contribute to positive neuroplasticity. A study involved the performance of C5 to C6 severely injured subjects in 12 visuospatial-motor training tasks (2–3 sessions per week). Subjects used a body-machine interface with a non-invasive inertial measurement unit to manipulate a 2D cursor while simultaneously moving both shoulders. The subjects’ upper body capacities were assessed before the start of training, during training, and one day after the end of training. MRI data were obtained before the start of training and within 2 days after the end of training. The results showed that these patients learned to control the body-machine interface using injury-free upper-body movements and that the practice improved their performance. Those trained in exercise demonstrated improved scores on manual muscle strength tests and showed increased isometric muscular strength measured in their shoulders and upper arms. Also, exercise-induced elevation of the value of the zonal anisotropy fraction in the left hemisphere by an average of 6.02% was observed. The modulation of axon diameter, myelin thickness, or correspondingly, the number of these atoms in local white matter indicated microstructural changes [108]. In a randomized controlled trial, 25 patients with chronic incomplete SCIs in the cervical, thoracic, or lumbar regions were randomly assigned to 10 sessions of combined corticospinal-motor neuronal stimulation (PCMS) and exercise or sham PCMS. Moreover, 180 pairs of stimuli were applied to the motor corticospinal volley evoked by TMS over the M1 and were timed to reach corticospinal-motor neuron synapses in the upper or lower limb muscles (depending on the level of injury) 1 to 2 ms before repetitive nerve potential excitation in neurons. Participants exercised for 45 min after the completion of all protocols. The study revealed that the duration required to finish all protocols of grading and redefined muscle strength, sensory and grip assessment subcomponents, and a 10 m walk test was reduced by an average of 20% per protocol. Despite the presence of PCMS and training, the amplitude of TMS evoked corticospinal responses and the magnitude of maximal voluntary contraction of target muscles were significantly increased by 40–50%. The behavioral and physiological effects of the PCMS training were maintained for 6 months, while those of the sham PCMS training did not show any changes after intervention. This suggests that stimulation may have played supplementary roles in maintaining the training effects [109]. Another paper displayed the relationship between white matter plasticity in training-induced sensory-motor recovery in preclinical and clinical SCIs. In addition, white matter plasticity in the form of oligodendrogenesis (mice) and increased myelin water fraction (humans) occurred in response to chronic rehabilitation, suggesting that regarding spontaneous recovery after SCIs, neurorehabilitation, or motor rehabilitation on a flat treadmill, motor control is not learned [110]. In another study, nine long-suffering spinal cord-paralyzed patients (6–24 years post-injury, age range 27–44 years) recorded movement-related brain potentials while trying to move their paralyzed toes. Their data were compared with spinal cord-intact participants of the same age and gender (10 were only prepared for the movement, the other 10 performed the movement). The SCI group had lower amplitudes of preparatory and motor potentials than the control group, according to the results. Moreover, the SCI group and the non-motor control group had similar topographic distributions of motor potentials and readiness potential in comparison [111]. Another clinical comparative study included 24 patients with complete and incomplete sensorimotor paralysis and tetraplegia (12 with NP and 13 without pain) and 31 healthy subjects. Functional MRIs were used to assess activation in the primary somatosensory and motor cortex during motor (active and passive wrist extension) and sensory (warm-up and brushing) tasks using the dorsal hand. Compared to healthy controls, task-related activation (i.e., motor or sensory) did not differ at the group level. However, based on Euclidean distance measurements, SCI patients showed a lateral shift in peak activity in the primary sensory and motor cortex (*p* < 0.05). In patients with neuropathic pain, the intensity of chronic pain was inversely proportional to the magnitude of the shift in the M1 during wrist extension activity. This finding suggests that NP is not associated with increased plasticity in motor and sensory tasks above the lesion level [112]. Finally, a longitudinal study analyzed brain volume changes following intensive lower limb training using virtual reality with tensor-based morphometry in nine patients with traumatic incomplete SCIs. MRI data were acquired over a four-week training period (16–20 training sessions) before and after. Before training, voxel-based morphometry and voxel-based cortical thickness measurements were used to assess baseline morphological differences in nine SCI patients compared to 14 healthy controls. Intensive virtual reality-based training for limb control significantly improved patients’ balance, walking speed, gait, and muscle strength. Sustained clinical improvement was confirmed at a follow-up of 3–4 months. Voxel-based morphometry of patients compared to controls showed a reduction in white matter volume in the brainstem and cerebellum, and voxel-based cortical thickness showed cortical thinning in the M1. Tensor-based morphometry indicated that the volume of the cerebellum and left middle temporal lobe increased significantly over time. Incomplete SCIs resulted in heightened volumes of the left middle temporal gyrus, occipital gyrus, left temporal pole, cuneiform gyrus, hippocampi, cerebellum, corpus callosum, and brainstem. SCI patients underwent structural plasticity at the cortical and brainstem levels through virtual reality-augmented training, as demonstrated in this study [113].

### 3.3. Discussion

Our review aimed to analyze the brain plasticity in patients with SCIs and its influence on the rehabilitation process. The studies included in this review have demonstrated that neuroplasticity can affect the sensory-motor network (M1, cingulate cortex, premotor cortex, and supplementary motor cortex) and that different protocols and rehabilitation interventions can activate this process in different ways. PAS and TMS significantly increase corticospinal excitability in patients with SCI who show good motor recovery. In addition, Hebbian stimulation leads to further improvements in walking speed and corticospinal function, and larger MEP size reflects residual corticospinal connectivity. Walking and forms of endurance training may also have a positive effect on cutaneous muscle reflexes, including increased reflex inhibition of the soleus muscle during walking and decreased clonus due to reduced reflex excitability [105,106,107]. Furthermore, in patients with SCI, visuospatial motor training has been found to induce changes in local white matter microstructure, such as axon diameter, myelin thickness, and axon number, and increase muscle strength. Instead, PMCS can increase the maximal voluntary contraction magnitude of target muscles, and behavioral and physiological effects persist after 6 months. Exercise rehabilitation training in humans with SCIs can elicit white matter plasticity in the form of increased myelin water content and oligodendrogenesis in mice [108,109,110]. SCI patients may have low brain potentials (when attempting to move a paralyzed toe) and the topographic distribution of motor potentials is similar to that of non-motorized individuals. In addition, this clinical population may show lateral shifts in peak activity in the primary motor and sensory cortices during motor and sensory tasks, suggesting that NP is not associated with increased plasticity in these specific tasks. Finally, intensive virtual reality training for limb control can significantly improve patients’ balance, walking speed, gait, muscle strength, and left middle temporal lobe and cerebellar volumes [111,112,113]. According to the scientific literature, corticospinal plasticity is believed to mediate functional recovery after SCIs and may facilitate the reorganization of the cortex. During motor learning, there is an increase in the plasticity of the motor maps and a greater proportion of cortical areas that are allocated to the learned task. For example, when humans learn to play the piano correctly, their finger expression tends to look more natural [114]. Motor learning in primates is believed to involve the acquisition of skilled behaviors due to the plasticity observed in motor maps, as there is no reorganization of cortical structures during repetitive unskilled tasks that do not require learning [115]. There are several mechanisms underlying motor map plasticity after motor learning [116], including changes in protein synthesis [117], dendritic remodeling [118], synapse formation [119], synaptic efficacy [120], and these can alter synaptic weight between pyramidal neurons and change representational boundaries [121,122]. Furthermore, the enhancement of minor but non-intersecting CST fragments could facilitate functional recuperation post-SCI. In humans, ipsilateral CST can provide functional adaptation to the acute dysfunction of contralateral corticospinal connections [123]. Additionally, brain imaging and reversible drug therapy following cervical corticospinal transection in primates demonstrated that dexterity recovery involves the activation of the bilateral motor cortex, but during chronic illness, it was discovered that the contralateral area was moved during the initial recovery [124]. Another possibility for restoring corticospinal input after SCI is the emergence of corticospinal collaterals on the reticulospinal nucleus of the brainstem [125] This can mediate functional recovery by bilaterally accessing the spinal cord circuitry caudal to the injury via the cortico-reticular-intrinsic spinal cord relay [51,126]. Species that have evolved from primitive protostomes to humans use the neural circuits in their spine to initiate lower limb movements [127,128]. These spinal circuits are surprisingly plastic and have been shown in the literature to remodel in a functionally significant way when some or all of the descending input is lost [129]. There is also evidence for the proposed use of new technologies and techniques, such as deep brain stimulation of midbrain motor centers (mesencephalic motor cortex), as therapeutic strategies to restore motor function after SCIs or stroke provoking plastic changes. Very promising results have been obtained in rodent models with a >80% spinal cord transection, and this technique resulted in acutely functional hindlimb walking and swimming movements [130,131]. The use of stem cell-based regenerative therapy is an innovative approach to inducing a new type of plasticity, which can be produced through SCIs. Both clinical and preclinical studies have reported significant therapeutic effects in both acute and subacute settings utilizing different cell types, including neural stem/progenitor cells, mesenchymal stem cells, and olfactory capsule cells. Multiple therapeutic mechanisms have been explained by researchers, such as transplanted cell-mediated neuronal replacement, remyelination, and trophic support, which further induce tissue preservation and enhance neuronal plasticity [132,133]. Typical beneficial effects of rehabilitation in stem cell therapy include the neuroprotection of host tissues [134,135], induction of cell differentiation into neurons and oligodendrocytes in grafts [136,137], regeneration of neurons and axons [138,139], and reorganization of the lumbar circuit [136,137,140,141], all of which enhance functional recovery and positive plastic changes. However, the use of this regenerative therapy remains medically controversial due to various concerns regarding side effects, such as fever, infection, sensory impairment, and muscle weakness [142], and the risk of tumor formation from embryonic and induced pluripotent stem cell-derived cells [143,144].

This scoping review had several strengths. It is based on evidence from only human SCI populations and randomized controlled trial studies with medium to large sample sizes. It includes an analysis of the neural reorganization during rehabilitation in SCI patients as well as some tests to detect evoked potentials. We have also identified data gaps in many areas, hopefully providing information for future research.

The main limitation of the present study is that few papers met the inclusion criteria, as we included only nine articles that explored brain plasticity in patients with SCI and its influence on the rehabilitation process, and only three of them focused on the cortical sensory-motor plasticity aspect. This, besides the heterogenous methodology and samples, prevents us from drawing robust evidence on this important topic. Three databases were also used and the articles were restricted by date, so it is possible that important evidence was omitted. Furthermore, the sample sizes varied, as some were large and some were small, and the parameters measured were different. Doctors, psychiatrists, neuropsychologists, and clinical researchers in the field of rehabilitation need to consider the potential and ability of our brain to reorganize itself by investigating new neural pathways and effective therapeutic routes to ensure rapid functional recovery and an adequate quality of life in these patients.

## 4. Conclusions

In conclusion, this review displays that SCI patients may experience plastic changes either spontaneously or as a result of specific neurorehabilitation training, which may lead to positive outcomes in functional recovery. Evidence from both traumatic and non-traumatic SCIs suggest that the adult CNS undergoes plasticity through various events. This is supported by clinical and experimental evidence. Furthermore, efficacy-based, pharmacological, and genetic approaches, alone or in combination, are increasingly effective in promoting plasticity. These results provide a good indication of whether it is possible to prevent unfavorable plasticity and at the same time promote beneficial changes, what type of post-injury training is most beneficial and when it is beneficial, why training interacts with untrained function, and how many pharmacological and genetic modification approaches can be used, at what doses, and in what combination they should be applied. Given the few studies included in our work, the conclusions that can currently be drawn are preliminary, and the current evidence requires further investigations especially in the areas previously mentioned, particularly in terms of when they should be administered and which combinations work synergistically.

## Figures and Tables

**Table 1:** A summary of evoked potentials and tests.

Evoked Potentials and Tests	Description
Motor Evoked Potential (MEP)	MEPs are signals originating from descending motor pathways or muscles, which are recorded after the stimulation of motor paths in the brain. MEPs triggered by transcranial magnetic stimulation of the human motor cortex provide information about corticospinal excitability during stimulation [88].
Somatosensory Evoked Potential (SEP)	SEPs are electrical signals that provide somatosensory information and are transmitted through two major pathways within the spinal cord: the spinal lemniscus system and the spinothalamic system. SEP monitors only the dorsal lemniscal system, which transmits mechanoreception and proprioception. The recording of SEPs typically begins at the upper or lower extremity nerves, which are then picked up by the surgeon upon completion of surgery. Different parts of the sensory pathway receive electrical energy from electrodes. Sensory pathways are characterized by the transmission of evoked potentials from electrodes to the cortex, where waveforms are recorded [89].
Contact Heat Evoked Potential (CHEP)	To induce a brain EEG response, this neuroelectrophysiological technique involves applying neo-conscious tissue onto skin surfaces through rapid temperature changes (70 °C/s) via a fiber transmission. By examining the cortical responses, which include N2 latency, P2 latency, and N2-P2 amplitude, it is possible to determine the function of peripheral small nerve fibers [90,91].
Quantitative Sensory Testing (QST)	The use of this testing method involves a systematic approach to psychophysical testing that measures sensory thresholds such as pain, touch, vibration, and temperature. It measures personal sensation based on direct feedback from patients. It tests sensory loss (hypoesthesia, hypoesthesia) sensory gain (hyperalgesia, hyperalgesia, allodynia) nociception, and nociception of different afferent nerve fibers and central pathways [92,93,94].
Laser Evoked Potential (LEP)	LEP is a result of the brain’s response to laser-radiated heat pulses, which also trigger Aδ nociceptors. It is the most commonly used approach to investigate the function of nociceptive pathways in NP patients. Aδ and C nociceptors are activated by laser-generated radiant heat pulses, which generate “late” brain potential that is dependent on the activation of adjacent Aδ fibers [95].

Legend: Motor Evoked Potential (MEP), Somatosensory Evoked Potential (SEP), Contact Heat Evoked Potential (CHEP), Quantitative Sensory Testing (QST), Laser Evoked Potential (LEP).

**Table 2:** Summary of studies included in the research.

Author	Aim	Treatment Period	Sample Size	Outcomes Measures	Main Findings	Study Limitations
Versace et al., 2017 [105]	To assess whether SCI patients show altered sensory-motor plasticity within the M1.	Not Specificated.	10 Subjects with chronic SCIs and 10 Healthy Volunteers.	PAS, TMS. SCIM.	The PAS protocol significantly increased corticospinal excitability within 30 min in healthy subjects and SCI patients with good motor recovery but not in SCI patients with poor functional recovery.	PAS may not be beneficial in SCI patients with poor recovery due to difficulty in finding the right hotspot and potential differences in conductivity between patients and healthy controls.
Jo et al., 2023 [106]	The study aimed to enhance corticospinal-motor neuronal synapses at multiple spinal levels through Hebbian plasticity, thereby promoting functional recovery in the legs and arms.	8 to14 weeks.	20 Participants with chronic SCIs.	ISNCSCI.	Participants with Hebbian stimulation showed improved walking speed, corticospinal function, grip, gait, and quality of life compared to sham stimulation, with further improvements observed after nine months.	Small sample to generalize the data.
Khan et al., 2016 [107]	To determine the neural plasticity of spinal reflexes after two contrasting forms of walking training in individuals with chronic, motor-incomplete SCI.	6 months	20 Participants.	EMG, Electodes.	Reflex excitability, a specific response to training, was found to improve walking function, with participants with lower reflex excitability showing higher walking speed and distance.	Participants’ biases may have influenced the results, but they likely did not prefer one type of training over another, as they were unaware of the best exercise intervention.
Gonzalez et al., 2016 [108]	This study aimed to assess the rehabilitation effects and changes in white matter microstructure in patients with high SCI after bilateral upper extremity motor skill training.	Participants performed the visuospatial-motor training task in 12 sessions of 1.5 h: 2–3 times a week for a total of 4–6 weeks.	5 Subjects and 14 Control Subjects.	MMT	Exercise training enhances shoulder and upper arm MMT scores, isometric muscle strength, and FA values of the left hemisphere cingulate, indicating local white matter microstructural changes.	No additional clinical assessment was performed in the study. Motor dysfunction, muscle weakness, muscle atrophy, and cortical atrophy were shown to progress further with immobilization following SCIs.
Jo et al., 2020 [109]	The aim was to enhance functional recovery by working on the remaining neural networks.	10 sessions in 2–3 weeks.	25 Individuals with SCIs.	GRASSP	The GRASSP and 10 m walking tests were reduced by 20% in all protocols, but corticospinal responses and muscle contraction amplitude increased by 40–50% after PCMS with or without exercise.	The effectiveness of neuromodulatory approaches in enhancing exercise effects is not fully understood due to limited studies combining exercise with sham neurostimulation and stimulus intensity.
Faw et al., 2021 [110]	To examine whether the system promotes white matter plasticity and recovery in chronic incomplete SCIs.	12 weeks (3 times/week).	20 Individuals with SCIs.	MRI	This study indicates that eccentricity-focused downhill rehabilitation enhances white matter plasticity and functional recovery in chronic SCIs through oligodendrogenesis in neuronal regions activated by the training approach.	Many people with SCIs were excluded from human trials for the following reasons: spinal hardware, claustrophobia, and motion artifacts.
Castro et al., 2013 [111]	Look for detectable changes in neuroplasticity immediately after trauma.	Non Specificated.	20 Patients.	Stimuli Tasks, EEG.	The study found that the SCI group had smaller preparation and movement potential amplitudes and a more similar topographic distribution of movement potentials compared to the exercise control group.	Not detected.
Jutzeler et al., 2015 [112]	The study aimed to investigate the relationship between cortical reorganization and NP after SCI.	Not Specificated.	57 Subjects (26 with NP and 31 Healthy Individuals).	FMR, Sensory Tasks, EI.	The results suggest that NP is not associated with increased plasticity in motor and sensory tasks above the lesion level.	The study focused on the base of the thumb using various sensory modalities, lacking reorganization responses and addressing heterogeneity in the SCI sample compared to Wrigley.
Villiger et al., 2015 [113]	This study used longitudinal MRI to assess structural brain plasticity induced by improved training in patients with chronic inflammatory SCI.	Between August 2010 and March 2012.	9 Patients with iSCI.	MRI, TBM.	TBM volume increases in various brain regions, particularly in patients with iSCIs, with significant improvements observed in the left middle temporal and occipital gyrus, hippocampus, cerebellum, corpus callosum, and brainstem.	Small sample size. The lack of an SCI control group for training with virtual reality means that the changes induced by the training in patients could have occurred not only as part of the training but also as part of the placebo effect.

Motor Evoked Potential (MEP), Body Machine Interface (BMI), Movement-Related Brain Potentials (MRBPs), Statistical Package for the social sciences (SPSS), Virtual Reality (VR), Cutaneomuscular Reflex (CMR), -- The right one is -- Legend: Spinal Cord Injury (SCI), Primary Motor Cortex (M1), Paired Associative Stimulation (PAS), Transcranial Magnetic Stimulation (TMS), Spinal Cord In-dependence Measure (SCIM), Fractional Anisotropy (FA), Manual Muscle Test (MMT), Graded and Redefined Assessment of Strength, Sensibility and Prehension (GRASSP), Corticospinal-motor Neuronal Stimulation (PCMS), Magnetic Resonance Imaging (MRI), Electroencephalography (EEG), International Standards for Neurological Classification of Spinal Cord Injury (ISNCSCI), Neuropathic Pain (NP), Functional Magnetic Resonance (FMR), Edinburgh Inventory (EI), Incomplete Spinal Cord Injury (iSCI), Tensor-Based Morphometry (TBM), Bipolar surface electromyograms (EMG).

## Data Availability

The data that support the findings of this study are not openly available due to reasons of sensitivity and are available from the corresponding author upon reasonable request.

## References

[1] Solstrand Dahlberg L., Becerra L., Borsook D., Linnman C. (2018). Brain changes after spinal cord injury, a quantitative meta-analysis and review. Neurosci. Biobehav. Rev..

[2] Fehlings M.G., Tetreault L.A., Wilson J.R., Kwon B.K., Burns A.S., Martin A.R., Hawryluk G., Harrop J.S. (2017). A Clinical Practice Guideline for the Management of Acute Spinal Cord Injury: Introduction, Rationale, and Scope. Glob. Spine J..

[3] Anderson K.D. (2004). Targeting recovery: Priorities of the spinal cord-injured population. J. Neurotrauma.

[4] Fawcett J.W., Curt A., Steeves J.D., Coleman W.P., Tuszynski M.H., Lammertse D., Bartlett P.F., Blight A.R., Dietz V., Ditunno J. (2007). Guidelines for the conduct of clinical trials for spinal cord injury as developed by the ICCP panel: Spontaneous recovery after spinal cord injury and statistical power needed for therapeutic clinical trials. Spinal Cord.

[5] Rabchevsky A.G., Kitzman P.H. (2011). Latest approaches for the treatment of spasticity and autonomic dysreflexia in chronic spinal cord injury. Neurotherapeutics.

[6] Hiersemenzel L.-P., Curt A., Dietz V. (2000). From spinal shock to spasticity: Neuronal adaptations to a spinal cord injury. Neurology.

[7] Basbaum A., Wall P. (1976). Chronic changes in the response of cells in adult cat dorsal horn following partial deafferentation: The appearance of responding cells in a previously nonresponsive region. Brain Res..

[8] Fawcett J. (2002). Repair of spinal cord injuries: Where are we, where are we going?. Spinal Cord.

[9] Fouad K., Tse A. (2008). Adaptive changes in the injured spinal cord and their role in promoting functional recovery. Neurol. Res..

[10] Liu C.N., Chambers W.W. (1958). Intraspinal sprouting of dorsal root axons; development of new collaterals and preterminals following partial denervation of the spinal cord in the cat. AMA Arch. Neurol. Psychiatry.

[11] Murray M., Goldberger M.E. (1974). Restitution of function and collateral sprouting in the cat spinal cord: The partially hemisected animal. J. Comp. Neurol..

[12] Bruehlmeier M., Dietz V., Leenders K.L., Roelcke U., Missimer J., Curt A. (1998). How does the human brain deal with a spinal cord injury?. Eur. J. Neurosci..

[13] Endo T., Spenger C., Tominaga T., Brené S., Olson L. (2007). Cortical sensory map rearrangement after spinal cord injury: fMRI responses linked to Nogo signalling. Brain.

[14] Kaas J.H., Qi H.-X., Burish M.J., Gharbawie O.A., Onifer S.M., Massey J.M. (2008). Cortical and subcortical plasticity in the brains of humans, primates, and rats after damage to sensory afferents in the dorsal columns of the spinal cord. Exp. Neurol..

[15] Darian-Smith C., Gilbert C.D. (1994). Axonal sprouting accompanies functional reorganization in adult cat striate cortex. Nature.

[16] Fouad K., Pedersen V., Schwab M.E., Brösamle C. (2001). Cervical sprouting of corticospinal fibers after thoracic spinal cord injury accompanies shifts in evoked motor responses. Curr. Biol..

[17] Girgis J., Merrett D., Kirkland S., Metz G.A.S., Verge V., Fouad K. (2007). Reaching training in rats with spinal cord injury promotes plasticity and task specific recovery. Brain.

[18] Raineteau O., Schwab M.E. (2001). Plasticity of motor systems after incomplete spinal cord injury. Nat. Rev. Neurosci..

[19] Florence S., Kaas J. (1995). Large-scale reorganization at multiple levels of the somatosensory pathway follows therapeutic amputation of the hand in monkeys. J. Neurosci..

[20] Pons T.P., Garraghty P.E., Ommaya A.K., Kaas J.H., Taub E., Mishkin M. (1991). Massive cortical reorganization after sensory deafferentation in adult macaques. Science.

[21] Raineteau O., Fouad K., Noth P., Thallmair M., Schwab M.E. (2001). Functional switch between motor tracts in the presence of the mAb IN-1 in the adult rat. Proc. Natl. Acad. Sci. USA.

[22] Aoki M., Fujito Y., Satomi H., Kurosawa Y., Kasaba T. (1986). The possible role of collateral sprouting in the functional restitution of corticospinal connections after spinal hemisection. Neurosci. Res..

[23] Ballermann M., Fouad K. (2006). Spontaneous locomotor recovery in spinal cord injured rats is accompanied by anatomical plasticity of reticulospinal fibers. Eur. J. Neurosci..

[24] Bareyre F.M., Kerschensteiner M., Raineteau O., Mettenleiter T.C., Weinmann O., Schwab M.E. (2004). The injured spinal cord spontaneously forms a new intraspinal circuit in adult rats. Nat. Neurosci..

[25] Carp J.S., Wolpaw J.R. (1994). Motoneuron plasticity underlying operantly conditioned decrease in primate H-reflex. J. Neurophysiol..

[26] Darian-Smith C. (2004). Primary afferent terminal sprouting after a cervical dorsal rootlet section in the macaque monkey. J. Comp. Neurol..

[27] Hagg T., Oudega M. (2006). Degenerative and spontaneous regenerative processes after spinal cord injury. J. Neurotrauma.

[28] Weidner N., Ner A., Salimi N., Tuszynski M.H. (2001). Spontaneous corticospinal axonal plasticity and functional recovery after adult central nervous system injury. Proc. Natl. Acad. Sci. USA.

[29] Jain N., Florence S.L., Qi H.-X., Kaas J.H. (2000). Growth of new brainstem connections in adult monkeys with massive sensory loss. Proc. Natl. Acad. Sci. USA.

[30] Vavrek R., Girgis J., Tetzlaff W., Hiebert G.W., Fouad K. (2006). BDNF promotes connections of corticospinal neurons onto spared descending interneurons in spinal cord injured rats. Brain.

[31] Kim B.G., Dai H.-N., McAtee M., Vicini S., Bregman B.S. (2006). Remodeling of synaptic structures in the motor cortex following spinal cord injury. Exp. Neurol..

[32] Li G.L., Farooque M., Isaksson J., Olsson Y. (2004). Changes in synapses and axons demonstrated by synaptophysin immunohistochemistry following spinal cord compression trauma in the rat and mouse. Biomed. Environ. Sci..

[33] Harvey P.J., Lucas-Osma A.M., Li Y., Lin S., Black S., Singla R., Fouad K., Fenrich K.K., Bennett D.J., Dai Y. (2006). Persistent sodium currents and repetitive firing in motoneurons of the sacrocaudal spinal cord of adult rats. J. Neurophysiol..

[34] Ceto S., Sekiguchi K.J., Takashima Y., Nimmerjahn A., Tuszynski M.H. (2020). Neural Stem Cell Grafts Form Extensive Synaptic Networks that Integrate with Host Circuits after Spinal Cord Injury. Cell Stem Cell.

[35] Oudega M., Perez M.A. (2012). Corticospinal reorganization after spinal cord injury. J. Physiol..

[36] Lemon R.N. (2008). Descending pathways in motor control. Annu. Rev. Neurosci..

[37] Bouvier J., Caggiano V., Leiras R., Caldeira V., Bellardita C., Balueva K., Fuchs A., Kiehn O. (2015). Descending Command Neurons in the Brainstem that Halt Locomotion. Cell.

[38] Filli L., Engmann A.K., Zörner B., Weinmann O., Moraitis T., Gullo M., Kasper H., Schneider R., Schwab M.E. (2014). Bridging the gap: A reticulo-propriospinal detour bypassing an incomplete spinal cord injury. J. Neurosci..

[39] Fouad K., Schnell L., Bunge M.B., Schwab M.E., Liebscher T., Pearse D.D. (2005). Combining schwann cell bridges and olfactory-ensheathing glia grafts with chondroitinase promotes locomotor recovery after complete transection of the spinal cord. J. Neurosci..

[40] Huntley G.W. (1997). Correlation between patterns of horizontal connectivity and the extend of short-term representational plasticity in rat motor cortex. Cereb. Cortex.

[41] Weiss D.S., Keller A. (1994). Specific patterns of intrinsic connections between representation zones in the rat motor cortex. Cereb. Cortex.

[42] Murray K.C., Nakae A., Stephens M.J., Rank M., D’Amico J., Harvey P.J., Li X., Harris R.L.W., Ballou E.W., Anelli R. (2010). Recovery of motoneuron and locomotor function after spinal cord injury depends on constitutive activity in 5-HT2C receptors. Nat. Med..

[43] Takeoka A., Vollenweider I., Courtine G., Arber S. (2014). Muscle spindle feedback directs locomotor recovery and circuit reorganization after spinal cord injury. Cell.

[44] Locke K.C., Randelman M.L., Hoh D.J., Zholudeva L.V., Lane M.A. (2022). Respiratory plasticity following spinal cord injury: Perspectives from mouse to man. Neural Regen Res..

[45] Rosenzweig E.S., Courtine G., Jindrich D.L., Brock J.H., Ferguson A.R., Strand S.C., Nout Y.S., Roy R.R., Miller D.M., Beattie M.S. (2010). Extensive spontaneous plasticity of corticospinal projections after primate spinal cord injury. Nat. Neurosci..

[46] Friedli L., Rosenzweig E.S., Barraud Q., Schubert M., Dominici N., Awai L., Nielson J.L., Musienko P., Nout-Lomas Y., Zhong H. (2015). Pronounced species divergence in corticospinal tract reorganization and functional recovery after lateralized spinal cord injury favors primates. Sci. Transl. Med..

[47] Belhaj-Saïf A., Cheney P.D., Sugiyama Y., Higo N., Yoshino-Saito K., Murata Y., Nishimura Y., Oishi T., Isa T., Umeda T. (2000). Plasticity in the distribution of the red nucleus output to forearm muscles after unilateral lesions of the pyramidal tract. J. Neurophysiol..

[48] Müllner A., Gonzenbach R.R., Weinmann O., Schnell L., Liebscher T., Schwab M.E. (2008). Lamina-specific restoration of serotonergic projections after Nogo-A antibody treatment of spinal cord injury in rats. Eur. J. Neurosci..

[49] Hilton B.J., Anenberg E., Harrison T.C., Boyd J.D., Murphy T.H., Tetzlaff W. (2016). Re-Establishment of Cortical Motor Output Maps and Spontaneous Functional Recovery via Spared Dorsolaterally Projecting Corticospinal Neurons after Dorsal Column Spinal Cord Injury in Adult Mice. J. Neurosci..

[50] Hollis E.R., Ishiko N., Yu T., Lu C.-C., Haimovich A., Tolentino K., Richman A., Tury A., Wang S.-H., Pessian M. (2016). Ryk controls remapping of motor cortex during functional recovery after spinal cord injury. Nat. Neurosci..

[51] Asboth L., Friedli L., Beauparlant J., Martinez-Gonzalez C., Anil S., Rey E., Baud L., Pidpruzhnykova G., Anderson M.A., Shkorbatova P. (2018). Cortico–reticulo–spinal circuit reorganization enables functional recovery after severe spinal cord contusion. Nat. Neurosci..

[52] Courtine G., Song B., Roy R.R., Zhong H., Herrmann J.E., Ao Y., Qi J., Edgerton V.R., Sofroniew M.V. (2008). Recovery of supraspinal control of stepping via indirect propriospinal relay connections after spinal cord injury. Nat. Med..

[53] Ruder L., Takeoka A., Arber S. (2016). Long-Distance Descending Spinal Neurons Ensure Quadrupedal Locomotor Stability. Neuron.

[54] Chen B., Li Y., Yu B., Zhang Z., Brommer B., Williams P.R., Liu Y., Hegarty S.V., Zhou S., Zhu J. (2018). Reactivation of Dormant Relay Pathways in Injured Spinal Cord by KCC2 Manipulations. Cell.

[55] Kinoshita M., Matsui R., Kato S., Hasegawa T., Kasahara H., Isa K., Watakabe A., Yamamori T., Nishimura Y., Alstermark B. (2012). Genetic dissection of the circuit for hand dexterity in primates. Nature.

[56] Beauparlant J., van den Brand R., Barraud Q., Friedli L., Musienko P., Dietz V., Courtine G. (2013). Undirected compensatory plasticity contributes to neuronal dysfunction after severe spinal cord injury. Brain.

[57] Jiang Y.-Q., Zaaimi B., Martin J.H. (2016). Competition with Primary Sensory Afferents Drives Remodeling of Corticospinal Axons in Mature Spinal Motor Circuits. J. Neurosci..

[58] Edgerton V.R., Tillakaratne N.J., Bigbee A.J., de Leon R.D., Roy R.R. (2004). Plasticity of the spinal neural circuitry after injury. Annu. Rev. Neurosci..

[59] Navarrete-Opazo A., Alcayaga J., Sepúlveda O., Rojas E., Astudillo C. (2017). Repetitive Intermittent Hypoxia and Locomotor Training Enhances Walking Function in Incomplete Spinal Cord Injury Subjects: A Randomized, Triple-Blind, Placebo-Controlled Clinical Trial. J. Neurotrauma.

[60] Pearse D.D., Pereira F.C., Marcillo A.E., Bates M.L., Berrocal Y.A., Filbin M.T., Bunge M.B. (2004). cAMP and Schwann cells promote axonal growth and functional recovery after spinal cord injury. Nat. Med..

[61] Williams R.R., Henao M., Pearse D.D., Bunge M.B. (2015). Permissive schwann cell graft/spinal cord interfaces for axon regeneration. Cell Transplant..

[62] Shih C.-H., Lacagnina M., Leuer-Bisciotti K., Pröschel C. (2014). Astroglial-derived periostin promotes axonal regeneration after spinal cord injury. J. Neurosci..

[63] Zhang S., Alvarez D.J., Sofroniew M.V., Deming T.J. (2015). Design and synthesis of nonionic copolypeptide hydrogels with reversible thermoresponsive and tunable physical properties. Biomacromolecules.

[64] Assinck P., Duncan G.J., Hilton B.J., Plemel J.R., Tetzlaff W. (2017). Cell transplantation therapy for spinal cord injury. Nat. Neurosci..

[65] Anderson K.D., Guest J.D., Dietrich W.D., Bunge M.B., Curiel R., Dididze M., Green B.A., Khan A., Pearse D.D., Saraf-Lavi E. (2017). Safety of Autologous Human Schwann Cell Transplantation in Subacute Thoracic Spinal Cord Injury. J. Neurotrauma.

[66] Lu P., Wang Y., Graham L., McHale K., Gao M., Wu D., Brock J., Blesch A., Rosenzweig E.S., Havton L.A. (2012). Long-distance growth and connectivity of neural stem cells after severe spinal cord injury. Cell.

[67] Lu P., Jones L., Snyder E., Tuszynski M. (2003). Neural stem cells constitutively secrete neurotrophic factors and promote extensive host axonal growth after spinal cord injury. Exp. Neurol..

[68] Kadoya K., Lu P., Nguyen K., Lee-Kubli C., Kumamaru H., Yao L., Knackert J., Poplawski G., Dulin J.N., Strobl H. (2016). Spinal cord reconstitution with homologous neural grafts enables robust corticospinal regeneration. Nat. Med..

[69] Bonner J.F., Connors T.M., Silverman W.F., Kowalski D.P., Lemay M.A., Fischer I. (2011). Grafted neural progenitors integrate and restore synaptic connectivity across the injured spinal cord. J. Neurosci..

[70] Abematsu M., Tsujimura K., Yamano M., Saito M., Kohno K., Kohyama J., Namihira M., Komiya S., Nakashima K. (2010). Neurons derived from transplanted neural stem cells restore disrupted neuronal circuitry in a mouse model of spinal cord injury. J. Clin. Investig..

[71] Fehlings M.G., Cadotte D.W., Fehlings L.N. (2011). A series of systematic reviews on the treatment of acute spinal cord injury: A foundation for best medical practice. J. Neurotrauma.

[72] Fisher A.R., Siegelman E.S. (2002). Magnetic resonance imaging: Techniques. Clin. Liver Dis..

[73] Freund P., Seif M., Weiskopf N., Friston K., Fehlings M.G., Thompson A.J., Curt A. (2019). MRI in traumatic spinal cord injury: From clinical assessment to neuroimaging biomarkers. Lancet Neurol..

[74] Weiskopf N., Suckling J., Williams G., Correia M.M., Inkster B., Tait R., Ooi C., Bullmore E.T., Lutti A. (2013). Quantitative multi-parameter mapping of R1, PD*, MT, and R2* at 3T: A multi-center validation. Front. Neurosci..

[75] Pfyffer D., Vallotton K., Curt A., Freund P. (2020). Tissue bridges predict neuropathic pain emergence after spinal cord injury. J. Neurol. Neurosurg. Psychiatry.

[76] Kyathanahally S.P., Azzarito M., Rosner J., Calhoun V.D., Blaiotta C., Ashburner J., Weiskopf N., Wiech K., Friston K., Ziegler G. (2021). Microstructural plasticity in nociceptive pathways after spinal cord injury. J. Neurol. Neurosurg. Psychiatry.

[77] Villanueva L., Le Bars D. (1995). The activation of bulbo-spinal controls by peripheral nociceptive inputs: Diffuse noxious inhibitory controls. Biol. Res..

[78] Djouhri L., Koutsikou S., Fang X., McMullan S., Lawson S.N. (2006). Spontaneous Pain, Both Neuropathic and Inflammatory, Is Related to Frequency of Spontaneous Firing in Intact C-Fiber Nociceptors. J. Neurosci..

[79] Hains B.C., Waxman S.G. (2007). Sodium channel expression and the molecular pathophysiology of pain after SCI. Prog. Brain Res..

[80] Detloff M.R., Fisher L.C., McGaughy V., Longbrake E.E., Popovich P.G., Basso D.M. (2008). Remote activation of microglia and pro-inflammatory cytokines predict the onset and severity of below-level neuropathic pain after spinal cord injury in rats. Exp. Neurol..

[81] Wiech K. (2016). Deconstructing the sensation of pain: The influence of cognitive processes on pain perception. Science.

[82] Hanna A.S., Filipp M., Travis B.J., Henry S.S., Idzikowski E.C., Magnuson S., Loh M.Y., Hellenbrand D.J. (2019). Differences in neuroplasticity after spinal cord injury in varying animal models and humans. Neural Regen. Res..

[83] Rao J.-S., Zhao C., Bao S.-S., Feng T., Xu M. (2022). MRI metrics at the epicenter of spinal cord injury are correlated with the stepping process in rhesus monkeys. Exp. Anim..

[84] Rao J.-S., Zhao C., Wei R.-H., Feng T., Bao S.-S., Zhao W., Tian Z., Liu Z., Yang Z.-Y., Li X.-G. (2022). Neural regeneration therapy after spinal cord injury induces unique brain functional reorganizations in rhesus monkeys. Ann. Med..

[85] Segal M.E., Ditunno J.F., Staas W.E. (1993). Interinstitutional agreement of individual functional independence measure (FIM) items measured at two sites on one sample of SCI patients. Spinal Cord.

[86] Bluvshtein V., Front L., Itzkovich M., Aidinoff E., Gelernter I., Hart J., Biering-Soerensen F., Weeks C., Laramee M.T., Craven C. (2010). SCIM III is reliable and valid in a separate analysis for traumatic spinal cord lesions. Spinal Cord.

[87] Itzkovich M., Gelernter I., Biering-Sorensen F., Weeks C., Laramee M.T., Craven B.C., Tonack M., Hitzig S.L., Glaser E., Zeilig G. (2007). The Spinal Cord Independence Measure (SCIM) version III: Reliability and validity in a multi-center international study. Disabil. Rehabil..

[88] Bestmann S., Krakauer J.W. (2015). The uses and interpretations of the motor-evoked potential for understanding behaviour. Exp. Brain Res..

[89] Cruccu G., Aminoff M., Curio G., Guerit J., Kakigi R., Mauguiere F., Rossini P., Treede R.-D., Garcia-Larrea L. (2008). Recommendations for the clinical use of somatosensory-evoked potentials. Clin. Neurophysiol..

[90] Lagerburg V., Bakkers M., Bouwhuis A., Hoeijmakers J.G., Smit A.M., Berg S.J.V.D., Boer I.H., Der Lee M.D.B., Kranendonk D., Reulen J.P. (2015). Contact heat evoked potentials: Normal values and use in small-fiber neuropathy. Muscle Nerve.

[91] Granovsky Y., Anand P., Nakae A., Nascimento O., Smith B., Sprecher E., Valls-Solé J. (2016). Normative data for Aδ contact heat evoked potentials in adult population. Pain.

[92] Horowitz S.H. (2009). Neuropathic pain: Is the emperor wearing clothes. Current Therapy in Pain.

[93] Weaver K.R., Griffioen M.A., Klinedinst N.J., Galik E., Duarte A.C., Colloca L., Resnick B., Dorsey S.G., Renn C.L. (2022). Quantitative Sensory Testing Across Chronic Pain Conditions and Use in Special Populations. Front. Pain Res..

[94] Mücke M., Cuhls H., Radbruch L., Baron R., Maier C., Tölle T., Treede R.-D., Rolke R. (2014). Quantitative sensory testing (QST). English version. Der Schmerz.

[95] Ditunno P.L., Patrick M., Stineman M., Ditunno J.F. (2008). Who wants to walk? Preferences for recovery after SCI: A longitudinal and cross-sectional study. Spinal Cord.

[96] Truini A., Panuccio G., Galeotti F., Maluccio M., Sartucci F., Avoli M., Cruccu G. (2010). Laser-evoked potentials as a tool for assessing the efficacy of antinociceptive drugs. Eur. J. Pain.

[97] Barbeau H., McCrea D.A., O’Donovan M.J., Rossignol S., Grill W.M., Lemay M.A. (1999). Tapping into spinal circuits to restore motor function. Brain Res. Rev..

[98] Wernig A., Müller S. (1992). Laufband locomotion with body weight support improved walking in persons with severe spinal cord injuries. Spinal Cord.

[99] Grillner S. (1985). Neurobiological Bases of Rhythmic Motor Acts in Vertebrates. Science.

[100] Sherrington C.S. (1910). Flexion-reflex of the limb, crossed extension-reflex, and reflex stepping and standing. J. Physiol..

[101] Rossignol S., Dubuc R., Gossard J.-P. (2006). Dynamic Sensorimotor Interactions in Locomotion. Physiol. Rev..

[102] Dietz V., Sinkjaer T. (2007). Spastic movement disorder: Impaired reflex function and altered muscle mechanics. Lancet Neurol..

[103] Pearson K.G. (2000). Neural Adaptation in the Generation of Rhythmic Behavior. Annu. Rev. Physiol..

[104] Dietz V. (2002). Proprioception and locomotor disorders. Nat. Rev. Neurosci..

[105] Versace V., Langthaler P.B., Höller Y., Frey V.N., Brigo F., Sebastianelli L., Saltuari L., Nardone R. (2017). Abnormal cortical neuroplasticity induced by paired associative stimulation after traumatic spinal cord injury: A preliminary study. Neurosci. Lett..

[106] Jo H.J., Kizziar E., Sangari S., Chen D., Kessler A., Kim K., Anschel A., Heinemann A.W., Mensh B.D., Awadalla S. (2023). Multisite Hebbian Plasticity Restores Function in Humans with Spinal Cord Injury. Ann. Neurol..

[107] Khan A.S., Patrick S.K., Roy F.D., Gorassini M.A., Yang J.F. (2016). Training-Specific Neural Plasticity in Spinal Reflexes after Incomplete Spinal Cord Injury. Neural Plast..

[108] Seáñez-González I., Pierella C., Farshchiansadegh A., Thorp E.B., Wang X., Parrish T., Mussa-Ivaldi F.A. (2016). Body-Machine Interfaces after Spinal Cord Injury: Rehabilitation and Brain Plasticity. Brain Sci..

[109] Jo H.J., Perez M.A. (2020). Corticospinal-motor neuronal plasticity promotes exercise-mediated recovery in humans with spinal cord injury. Brain.

[110] Faw T.D., Lakhani B., Schmalbrock P., Knopp M.V., Lohse K.R., Kramer J.L., Liu H., Nguyen H.T., Phillips E.G., Bratasz A. (2021). Eccentric rehabilitation induces white matter plasticity and sensorimotor recovery in chronic spinal cord injury. Exp. Neurol..

[111] Castro A., Díaz F., Sumich A. (2013). Long-term neuroplasticity in spinal cord injury patients: A study on movement-related brain potentials. Int. J. Psychophysiol..

[112] Jutzeler C.R., Freund P., Huber E., Curt A., Kramer J.L. (2015). Neuropathic Pain and Functional Reorganization in the Primary Sensorimotor Cortex After Spinal Cord Injury. J. Pain.

[113] Villiger M., Grabher P., Hepp-Reymond M.-C., Kiper D., Curt A., Bolliger M., Hotz-Boendermaker S., Kollias S., Eng K., Freund P. (2015). Relationship between structural brainstem and brain plasticity and lower-limb training in spinal cord injury: A longitudinal pilot study. Front. Hum. Neurosci..

[114] Pascual-Leone A., Nguyet D., Brasil-Neto J.P., Cammarota A., Seidel O., Carius D., Kenville R., Ragert P., Stöckel T., Carroll T.J. (1995). Modulation of muscle responses evoked by transcranial magnetic stimulation during the acquisition of new fine motor skills. J. Neurophysiol..

[115] Plautz E.J., Milliken G.W., Nudo R.J. (2000). Effects of Repetitive Motor Training on Movement Representations in Adult Squirrel Monkeys: Role of Use versus Learning. Neurobiol. Learn. Mem..

[116] Papale A.E., Hooks B.M. (2017). Circuit Changes in Motor Cortex During Motor Skill Learning. Neuroscience.

[117] Kleim J.A., Bruneau R., Calder K., Pocock D., VandenBerg P.M., MacDonald E., Monfils M.H., Sutherland R.J., Nader K. (2003). Functional Organization of Adult Motor Cortex Is Dependent upon Continued Protein Synthesis. Neuron.

[118] Xu T., Yu X., Perlik A.J., Tobin W.F., Zweig J.A., Tennant K., Jones T., Zuo Y. (2009). Rapid formation and selective stabilization of synapses for enduring motor memories. Nature.

[119] Kleim J.A., Barbay S., Cooper N.R., Hogg T.M., Reidel C.N., Remple M.S., Nudo R.J. (2002). Motor Learning-Dependent Synaptogenesis Is Localized to Functionally Reorganized Motor Cortex. Neurobiol. Learn. Mem..

[120] Monfils M.-H., Teskey G. (2004). Skilled-learning-induced potentiation in rat sensorimotor cortex: A transient form of behavioural long-term potentiation. Neuroscience.

[121] Hess G., Donoghue J.P., Sato D., Yamashiro K., Onishi H., Yasuhiro B., Shimoyama Y., Maruyama A., Mang C.S., Snow N.J. (1994). Long-term potentiation of horizontal connections provides a mechanism to reorganize cortical motor maps. J. Neurophysiol..

[122] Rioult-Pedotti M.-S., Friedman D., Donoghue J.P. (2000). Learning-Induced LTP in Neocortex. Science.

[123] Strens L.H., Fogelson N., Shanahan P., Rothwell J.C., Brown P. (2003). The Ipsilateral Human Motor Cortex Can Functionally Compensate for Acute Contralateral Motor Cortex Dysfunction. Curr. Biol..

[124] Nishimura Y., Onoe H., Morichika Y., Perfiliev S., Tsukada H., Isa T. (2007). Time-Dependent Central Compensatory Mechanisms of Finger Dexterity After Spinal Cord Injury. Science.

[125] Zörner B., Bachmann L.C., Filli L., Kapitza S., Gullo M., Bolliger M., Starkey M.L., Röthlisberger M., Gonzenbach R.R., Schwab M.E. (2014). Chasing central nervous system plasticity: The brainstem’s contribution to locomotor recovery in rats with spinal cord injury. Brain.

[126] Matsuyama K., Mori F., Nakajima K., Drew T., Aoki M., Mori S. (2004). Locomotor role of the corticoreticular-reticulospinal-spinal in-terneuronal system. Prog. Brain Res..

[127] Dimitrijevic M.R., Gerasimenko Y., Pinter M.M. (1998). Evidence for a Spinal Central Pattern Generator in Humans. Ann. N. Y. Acad. Sci..

[128] Nadeau S., Jacquemin G., Fournier C., Lamarre Y., Rossignol S. (2009). Spontaneous Motor Rhythms of the Back and Legs in a Patient With a Complete Spinal Cord Transection. Neurorehabilit. Neural Repair.

[129] Martinez M., Brown A. (2019). From cortex to cord: Motor circuit plasticity after spinal cord injury. Neural Regen. Res..

[130] Fluri F., Malzahn U., Homola G.A., Schuhmann M.K., Kleinschnitz C., Volkmann J. (2017). Stimulation of the mesencephalic locomotor region for gait recovery after stroke. Ann. Neurol..

[131] Bachmann L.C., Matis A., Lindau N.T., Felder P., Gullo M., Schwab M.E. (2013). Deep Brain Stimulation of the Midbrain Locomotor Region Improves Paretic Hindlimb Function After Spinal Cord Injury in Rats. Sci. Transl. Med..

[132] Ahuja C.S., Fehlings M. (2016). Concise Review: Bridging the Gap: Novel Neuroregenerative and Neuroprotective Strategies in Spinal Cord Injury. Stem Cells Transl. Med..

[133] Nakamura M., Okano H. (2013). Cell transplantation therapies for spinal cord injury focusing on induced pluripotent stem cells. Cell Res..

[134] Massoto T.B., Santos A.C.R., Ramalho B.S., Almeida F.M., Martinez A.M.B., Marques S.A. (2019). Mesenchymal stem cells and treadmill training enhance function and promote tissue preservation after spinal cord injury. Brain Res..

[135] Younsi A., Zheng G., Scherer M., Riemann L., Zhang H., Tail M., Hatami M., Skutella T., Unterberg A., Zweckberger K. (2020). Treadmill training improves survival and differentiation of transplanted neural precursor cells after cervical spinal cord injury. Stem Cell Res..

[136] Hwang D.H., Shin H.Y., Kwon M.J., Choi J.Y., Ryu B.-Y., Kim B.G. (2014). Survival of Neural Stem Cell Grafts in the Lesioned Spinal Cord Is Enhanced by a Combination of Treadmill Locomotor Training via Insulin-Like Growth Factor-1 Signaling. J. Neurosci..

[137] Tashiro S., Nishimura S., Iwai H., Sugai K., Zhang L., Shinozaki M., Iwanami A., Toyama Y., Liu M., Okano H. (2016). Functional Recovery from Neural Stem/Progenitor Cell Transplantation Combined with Treadmill Training in Mice with Chronic Spinal Cord Injury. Sci. Rep..

[138] Sachdeva R., Theisen C.C., Ninan V., Twiss J.L., Houlé J.D. (2015). Exercise dependent increase in axon regeneration into peripheral nerve grafts by propriospinal but not sensory neurons after spinal cord injury is associated with modulation of regeneration-associated genes. Exp. Neurol..

[139] Theisen C.C., Sachdeva R., Austin S., Kulich D., Kranz V., Houle J.D. (2017). Exercise and Peripheral Nerve Grafts as a Strategy To Promote Regeneration after Acute or Chronic Spinal Cord Injury. J. Neurotrauma.

[140] Takeoka A., Jindrich D.L., Muñoz-Quiles C., Zhong H., Brand R.v.D., Pham D.L., Ziegler M.D., Ramón-Cueto A., Roy R.R., Edgerton V.R. (2011). Axon Regeneration Can Facilitate or Suppress Hindlimb Function after Olfactory Ensheathing Glia Transplantation. J. Neurosci..

[141] Sun T., Ye C., Zhang Z., Wu J., Huang H. (2013). Cotransplantation of Olfactory Ensheathing Cells and Schwann Cells Combined with Treadmill Training Promotes Functional Recovery in Rats with Contused Spinal Cords. Cell Transplant..

[142] Zhang L., Fan X., Wang J.-Z., Lin X.-M. (2017). Stem cell transplantation for spinal cord injury: A meta-analysis of treatment effectiveness and safety. Neural Regen. Res..

[143] Tsuji O., Sugai K., Yamaguchi R., Tashiro S., Nagoshi N., Kohyama J., Iida T., Ohkubo T., Itakura G., Isoda M. (2018). Concise Review: Laying the Groundwork for a First-In-Human Study of an Induced Pluripotent Stem Cell-Based Intervention for Spinal Cord Injury. Stem Cells.

[144] Lee A.S., Tang C., Rao M.S., Weissman I.L., Wu J.C. (2013). Tumorigenicity as a clinical hurdle for pluripotent stem cell therapies. Nat. Med..

